# Human Herpesvirus 8 (HHV8) Sequentially Shapes the NK Cell Repertoire during the Course of Asymptomatic Infection and Kaposi Sarcoma

**DOI:** 10.1371/journal.ppat.1002486

**Published:** 2012-01-12

**Authors:** Stéphanie Dupuy, Marion Lambert, David Zucman, Siméon-Pierre Choukem, Sara Tognarelli, Cécile Pages, Céleste Lebbé, Sophie Caillat-Zucman

**Affiliations:** 1 Institut National de la Santé et de la Recherche Médicale (INSERM), U986, Hôpital St-Vincent de Paul; Université Paris Descartes, Faculté de Médecine, Paris, France; 2 Hôpital Foch, Service de Médecine Interne, Suresnes, France; 3 AP-HP, Hôpital Saint-Louis, Service d'Endocrinologie; Université Paris Diderot, Faculté de Médecine, Paris, France; 4 AP-HP, Hôpital Saint-Louis, Service de Dermatologie; Université Paris Diderot, INSERM U976 Skin Research Center, Paris, France; University of North Carolina at Chapel Hill, United States of America

## Abstract

The contribution of innate immunity to immunosurveillance of the oncogenic Human Herpes Virus 8 (HHV8) has not been studied in depth. We investigated NK cell phenotype and function in 70 HHV8-infected subjects, either asymptomatic carriers or having developed Kaposi's sarcoma (KS). Our results revealed substantial alterations of the NK cell receptor repertoire in healthy HHV8 carriers, with reduced expression of NKp30, NKp46 and CD161 receptors. In addition, down-modulation of the activating NKG2D receptor, associated with impaired NK-cell lytic capacity, was observed in patients with active KS. Resolution of KS after treatment was accompanied with restoration of NKG2D levels and NK cell activity. HHV8-latently infected endothelial cells overexpressed ligands of several NK cell receptors, including NKG2D ligands. The strong expression of NKG2D ligands by tumor cells was confirmed in situ by immunohistochemical staining of KS biopsies. However, no tumor-infiltrating NK cells were detected, suggesting a defect in NK cell homing or survival in the KS microenvironment. Among the known KS-derived immunoregulatory factors, we identified prostaglandin E2 (PGE2) as a critical element responsible for the down-modulation of NKG2D expression on resting NK cells. Moreover, PGE2 prevented up-regulation of the NKG2D and NKp30 receptors on IL-15-activated NK cells, and inhibited the IL-15-induced proliferation and survival of NK cells. Altogether, our observations are consistent with distinct immunoevasion mechanisms that allow HHV8 to escape NK cell responses stepwise, first at early stages of infection to facilitate the maintenance of viral latency, and later to promote tumor cell growth through suppression of NKG2D-mediated functions. Importantly, our results provide additional support to the use of PGE2 inhibitors as an attractive approach to treat aggressive KS, as they could restore activation and survival of tumoricidal NK cells.

## Author Summary

Natural Killer (NK) cells are part of the innate immune response against virus infections and tumors. Their activation is the net result of signals emanating from a panel of inhibitory and activating receptors recognizing specific ligands on target cells. Human Herpes Virus 8 (HHV8) is an oncogenic virus responsible of Kaposi Sarcoma (KS), a multifocal angiogenic tumor. How NK cells contribute to the control of infection by HHV8 infection and development of KS, is unclear. In this paper, we show different strategies used by HHV8 to escape NK cell response. Patients with asymptomatic infection or KS have down-modulated expression of NKp30, NKp46 and CD161 receptors. In addition, patients with active KS show additional down-modulation of the NKG2D activating receptor, associated with impaired NK-cell cytotoxicity against target cells. Resolution of KS correlates with regained NKG2D expression and cytotoxic function. We present evidence that down-modulation of NKG2D is mediated by inflammatory prostaglandin E2 (PGE2), known to be released by KS cells, and show that PGE2 acts by preventing IL-15-mediated activation of NK cells. These results strongly support the use of PGE2 inhibitors as an attractive approach to treat active KS.

## Introduction

Human herpesvirus 8 (HHV8), although known as Kaposi's sarcoma-associated herpesvirus (KSHV), is a γ herpes virus able to establish a predominantly latent, life-long infection in host's monocytes, dendritic cells (DCs), B lymphocytes, and endothelial cells. HHV8 is the etiological agent of Kaposi's sarcoma (KS), a multifocal angiogenic tumor consisting of spindle-shaped cells of endothelial origin and infiltrating leukocytes [1], [2]. HHV8 lytic cycle generally occurs following primary infection, and rapidly the virus enters the latent state. Reactivation leads to the initiation of the lytic cycle, which is necessary for virus propagation and survival. Within KS lesions, HHV8 infection is predominantly latent. KS is the most common neoplasm in untreated AIDS patients. It also occurs in immunosuppressed organ transplant recipients, and in some African or Mediterranean populations in the absence of overt immunosuppression (classical KS). The marked decline in the incidence of AIDS-KS since the advent of antiretroviral therapy (ART), and the frequent resolution of transplant-related KS after reduction of immunosuppression, highlight the key role of cellular immune responses in the control of HHV8 infection. We and others recently demonstrated the crucial role of HHV8-specific cytotoxic T lymphocytes (CTL) in controlling HHV8 replication, preventing malignancies in latently infected subjects, and conferring genuine resistance to persistent infection [3], [4]. The multiple mechanisms elaborated by herpesviruses to escape immune responses prompted us to explore further other immune cells involved in the control of HHV8 infection.

NK cells play a key role in early control of viral infections, through direct lysis of infected cells and secretion of cytokines controlling viral replication. NK cells also influence specific T-cell priming through direct cross talk with DCs, and thus participate to the establishment of antiviral adaptive responses [5]–[7]. NK cells are able to prevent and control the development and dissemination of tumors [8]. NK cells are therefore likely to represent critical obstacles HHV8 needs to effectively overcome, not only very early during infection prior to de novo viral gene transcription, but also later for the maintenance of persistent infection and establishment of tumors. NK cell activity is tightly regulated by a fine balance between activating and inhibitory signals [9]. The latter are mostly generated by the binding of HLA-1 molecules to the Killer cell Immunoglobulin-like Receptors (KIRs) and CD94/NKG2A, which guarantees that healthy self cells will be spared from NK cell-mediated lysis. Activating receptors, in particular NKG2D and the natural cytotoxicity receptors (NCR) NKp30, NKp44 and NKp46, detect the presence of stress-induced or infectious non-self ligands on abnormal cells. Other receptors, such as CD94/NKG2C, DNAM-1, NKp80, 2B4 and CD161, can modulate NK cell effector functions.

HHV8 has exploited several evasion mechanisms to avoid immune recognition [10]. In particular, the K3 and K5 ubiquitin ligases, expressed during the early lytic cycle, downregulate HLA-1 molecules on infected cells to avoid virus-specific CTL recognition [11]. This will at the same time sensitize the virus to missing-self recognition by NK cells, unless other evasion mechanisms operate simultaneously. Interestingly, K5 also downmodulates NKG2D ligands and the ICAM-1 adhesion molecule [12], thus helping HHV8 to evade NK cell surveillance in the early phase of HHV8 infection before establishing latency, and later during reactivation and viral replication. NK cells from HIV-viremic AIDS patients with active KS have a decreased cytolytic capacity against HHV8-infected body cavity B-cell lymphoma (BCBL-1) cells [13]. However, little is known about how HHV8 by itself, in the absence of HIV infection, prevents NK cells from killing latently-infected endothelial cells and KS tumor cells.

To address these questions, we investigated NK cell phenotype and functions in a large series of HHV8-infected subjects. Our data revealed substantial alterations in the expression of several NK cell receptors, even in asymptomatic HHV8 carriers. In addition, NK cells from patients with active KS showed a significant decrease of NKG2D expression, which was associated with impaired cytotoxic capacity. We identified PGE2, a known tumor-derived inflammatory molecule, as a factor responsible for NKG2D down-modulation, and for inhibition of IL-15-mediated activation and survival of NK cells. These observations are consistent with sequential immunoevasion mechanisms that may allow HHV8 to escape NK cell recognition at early stages of infection in order to establish latency, and later to promote tumor cell growth. Importantly, they give further support to the idea that PGE2 inhibition based therapy might provide an effective way to treat the active KS lesions.

## Results

### HHV8 infection modifies the NK cell phenotype

We addressed the putative influence of HHV8 infection on NK cell phenotype in a cohort of 70 HHV8-infected individuals, including 25 asymptomatic carriers (HHV8+ KS−) and 45 KS patients (HHV8+ KS+) in comparison with 45 HHV8-negative controls, all sub-grouped according to the presence or absence of HIV co-infection. To avoid any confounding effect of HIV replication on the NK cell repertoire, all HIV+ subjects from the different subgroups were HIV-aviremic, and were matched for age, CD4 T cell count, CD4 nadir, and duration of disease and ART.

**Figure 1:**
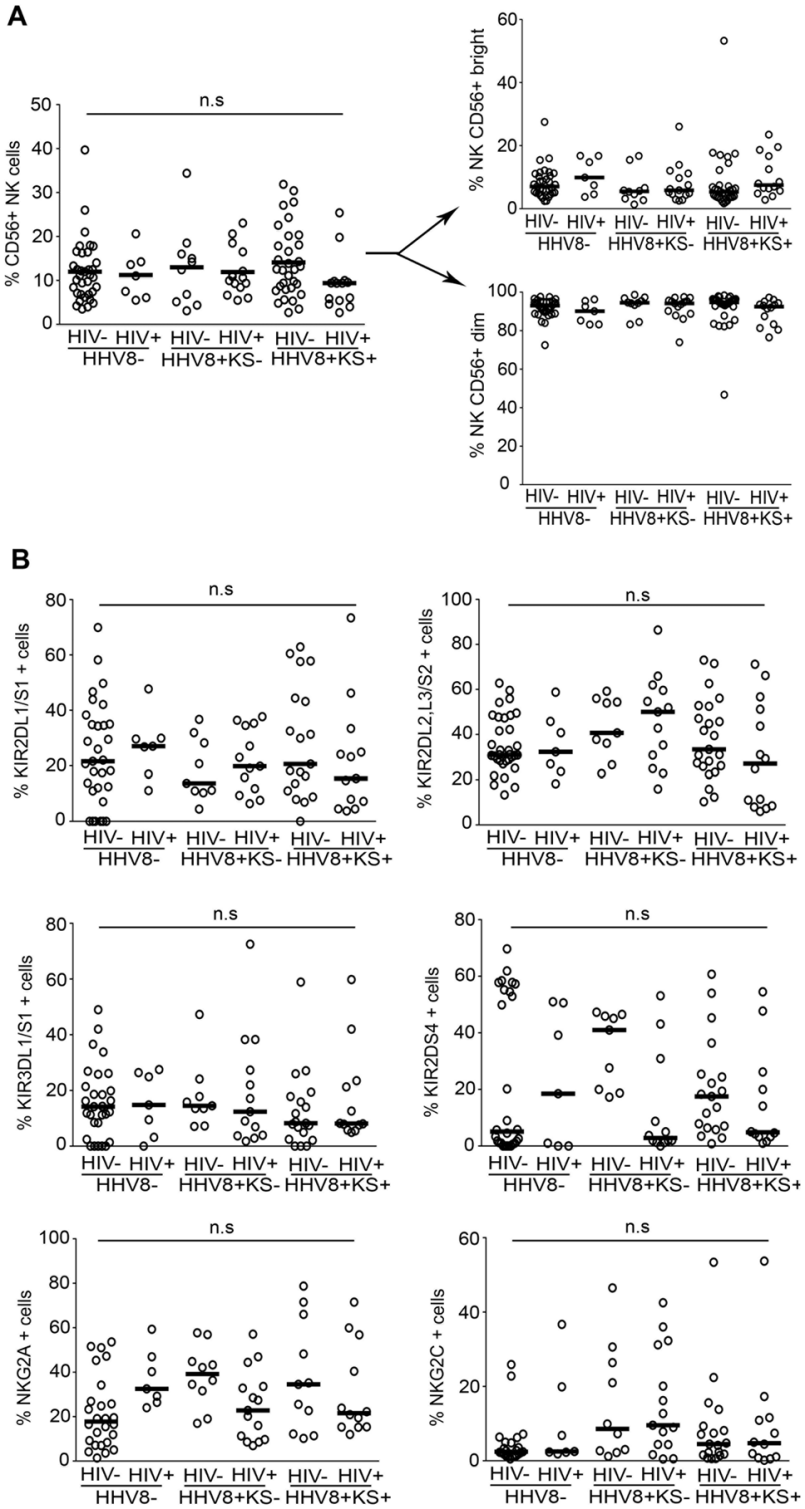
NK cell subset distribution in HHV8-infected subjects and controls. Summary graphs of statistical dot plots with medians (horizontal black bars) in the different study groups showing: (A) the percentage of CD3−CD56+ NK cells among PBMCs and the proportion of CD56bright and CD56 dim among NK cells; (B) the percentage of CD3−CD56+ NK cells expressing HLA class I-specific NK cell receptors. P values were not significant (n.s) after adjustment for multiple comparisons (Kruskall-Wallis test with Dunn's post test).

No gross abnormality in NK cell distribution was observed in the different patient groups, with levels of total NK cells, and relative proportions of CD56bright and CD56dim NK cell subpopulations being comparable to those in controls (Figure 1a). Accumulation of dysfunctional CD56-negative NK cells was recently reported in HIV-viremic patients, with suppression of HIV-viremia upon ART restoring normal proportions of CD56+ NK cells [14]–[16]. In line with the fact that all study subjects were HIV-aviremic, we did not observed any abnormal expansion of CD56-negative NK cells in HHV8-infected patients (Figure S1).

We next analyzed HLA class 1-specific NK cell receptors, including those belonging to the KIR family (KIR2DL1/S1, KIR2DL2/L3/S2, KIR3DL1/S1, KIR2DS4), and the HLA-E-specific CD94/NKG2A and CD94/NKG2C receptors (Figure 1b). Although large inter-individual variations were observed, the mean percentage of cells expressing individual KIRs or NKG2A was overall similar in the different study groups. The fraction of NKG2C+ NK cells varied within a wide range in infected patients (<0.1% to 54%), but the difference with uninfected controls was not statistically significant. CMV seropositivity has been associated with high frequencies of NKG2C+ NK cells [17], [18]. We thus wondered if the enrichment of NKG2C+ NK cells in some HHV8-infected patients was related to CMV co-infection. Notably, 68 out of the 70 HHV8- and/or HIV-infected patients were CMV IgG positive, and the two remaining CMV IgG-negative patients had very low NKG2C+ NK cell frequency (1.56% and 0.25%, respectively). Therefore, it is likely that previous CMV infection has driven expansion of NKG2C+ NK cells in HIV- or HHV8-infected patients.

**Figure 2:**
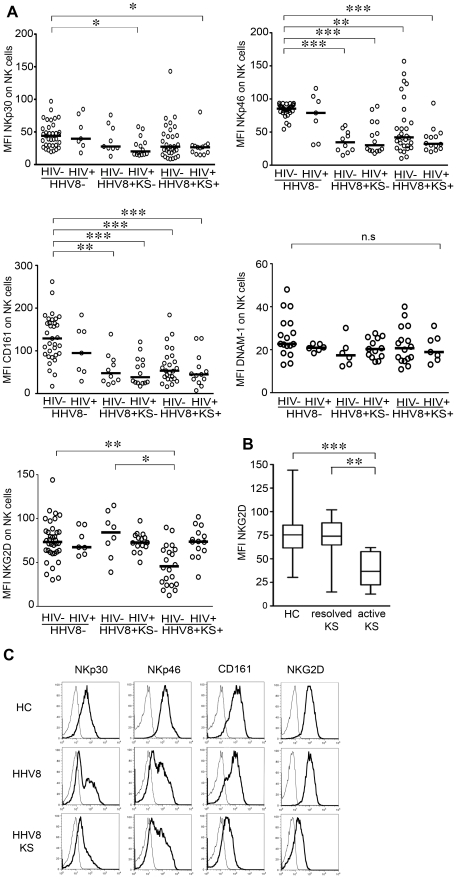
Alterations of NK cell receptor expression in HHV8-infected individuals. (A) Summary graphs of statistical dot plots with medians (horizontal black bars) showing expression (mean fluorescence intensity, MFI) of NKp30, NKp46, CD161, DNAM-1 and NKG2D receptors on gated CD3−CD56+ NK cells in the different study groups. Cells were analyzed on a FACSCalibur flow cytometer. P values<0.05 after adjustment for multiple comparisons (Kruskall-Wallis test with Dunn's post test) are indicated. * P<0.01, ** P<0.005, *** P<0.0001. (B) Box and whisker plots showing the median and 25–75^th^ percentiles of NKG2D expression in healthy controls (n = 35), patients with active KS (n = 10, all classical HIV- KS) and patients with resolved KS (n = 35, including 21 classical HIV- KS and 14 HIV+ KS). Horizontal bars indicate the minimal and maximal values. ** P<0.01, *** P<0.001. (C) Representative flow cytometry analysis in healthy controls (upper panel), asymptomatic HHV8 carriers (middle panel) and patients with active classical KS (lower panel). Control isotypes are shown as dotted lines.

We next studied expression of NK cell receptors that recognize virus-associated or stressed-induced molecules (Figure 2a). Levels of NKp30, NKp46 and CD161 were significantly decreased in HHV8-infected patients compared to controls, whatever the presence or absence of KS, indicating that HHV8 is able to skew the NK cell receptor repertoire in otherwise healthy individuals, before overt tumor transformation. DNAM-1 expression was not significantly different between the groups. NKp44 was never detected (not shown). Notably, the profile of NKG2D expression was clearly distinct from that of other receptors, as NKG2D levels were decreased in patients with classical KS (HIV− HHV8+ KS+), but neither in HIV+ KS+ patients nor in asymptomatic HHV8-infected subjects (HHV8+ KS−). Because all HIV+ KS patients were HIV-aviremic upon ART for more than 1 year and showed complete clinical remission of KS at time of study, we wondered if NKG2D expression was correlated with the KS activity. Indeed, NKG2D levels were twofold lower in patients with active KS (all classical KS) than in patients with resolved lesions or in healthy controls (Figure 2b,c). By contrast, expression of other receptors was similarly decreased in KS patients, whatever disease activity (data not shown).

**Figure 3:**
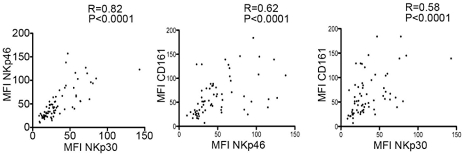
Coordinate decrease of NKp30, NKp46 and CD161 expression in HHV8-infected individuals. Positive correlations between the proportions of NK cells expressing the indicated receptors in HHV8-infected individuals. Spearman rank correlation (r) and P values are indicated.

The variable degrees of NK cell receptor modifications among patients prompted us to examine whether there was any correlation between phenotypic changes. We observed highly significant correlations between expression levels of NKp30, NKp46 and CD161, the three receptors downmodulated in asymptomatic HHV8-infected subjects (Figure 3), suggesting that a common mechanism sustained these alterations. At contrast, there was no correlation between expression of any of these receptors and expression of NKG2D.

Taken together, these results show that HHV8 infection selectively imprints the NK cell receptor repertoire even at an asymptomatic stage, with a coordinate decrease in NKp30, NKp46 and CD161 expression. At a more advanced stage, a specific down-modulation of NKG2D occurs in patients progressing to KS, which is likely mediated by a distinct, KS-specific mechanism.

### Phenotype of HHV8-infected endothelial cells

**Figure 4:**
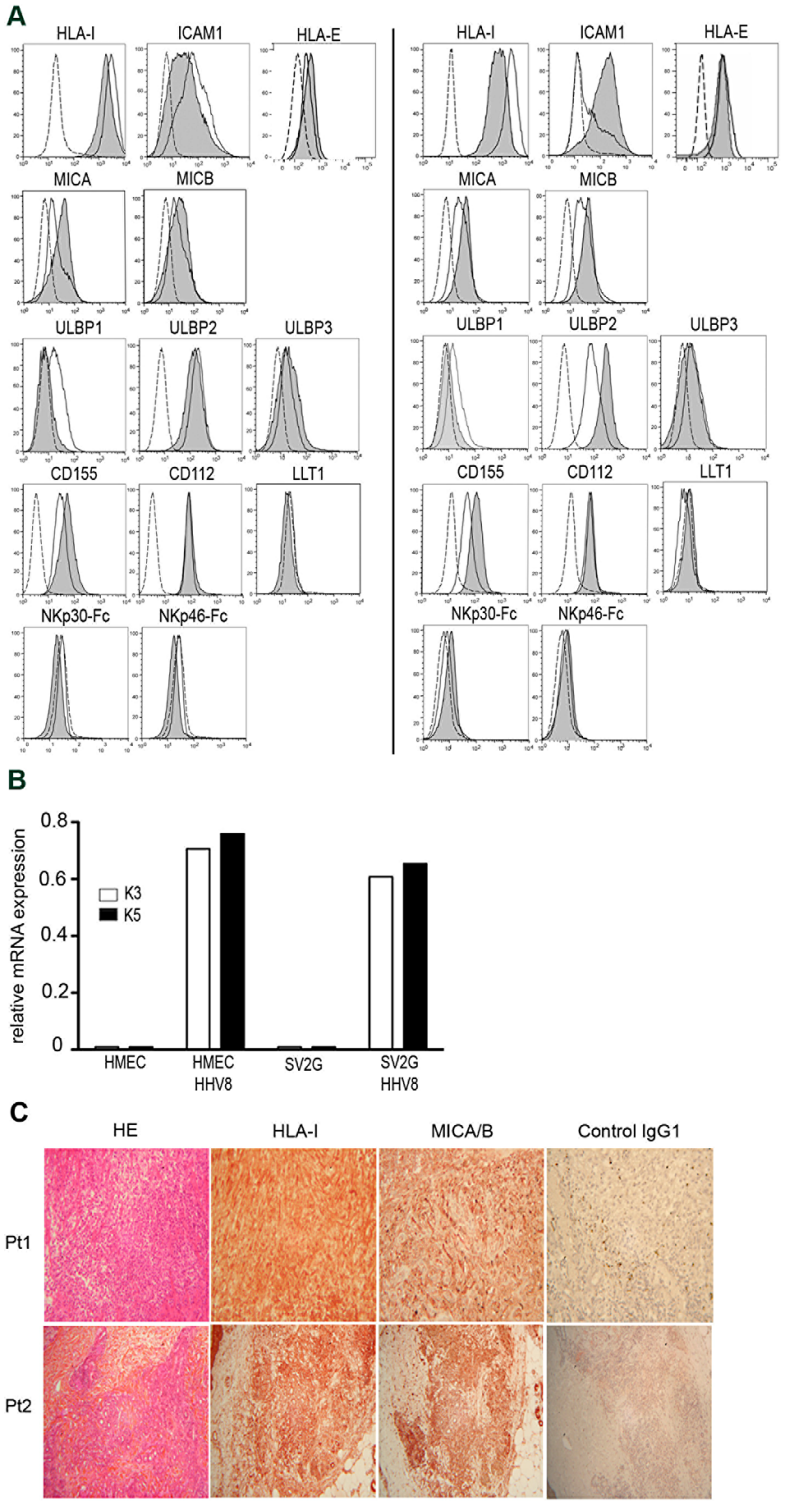
Expression of ligands for NK cell receptors on HHV8-infected cells. (A) Expression of the indicated ligand was measured by flow cytometry on uninfected (open histograms) and HHV8-infected (gray histograms) HMEC cells (left panels) and KS-derived cells (right panels). Dotted lines represent staining with control isotypes. Histograms are representative of 3–6 independent experiments performed in each cell line. B) K3 and K5 mRNA levels in uninfected or HHV8-infected HMEC and KS-derived cells. Results show the expression level of the K3 and K5 transcripts relative to glyceraldehyde-3-phosphate dehydrogenase (GAPDH) mRNA used as endogenous control, and represent the mean of 2 independent experiments. C) Consecutive sections of paraffin-embedded KS biopsies were stained with antibodies specific for HLA-1 (W6/32), MICA/B (SR99), or isotype control. Representative staining in 2 patients with classical KS out of 5 studied is shown. Staining with hematoxylin-eosin (HE) shows characteristic fascicles of spindle-shaped tumor cells forming the walls of slit-like vascular spaces, associated with mononuclear cell infiltrates. Original magnification x10 (Pt1), x40 (Pt2).

As a first step to study the molecular interactions underlying NK cell recognition of infected cells, we analyzed the phenotype of HHV8-infected endothelial cells, which are considered as one of the precursors of KS tumor cells. Primary infection of the microvascular endothelial cell line HMEC with a recombinant virus, rKSHV.152, expressing the green fluorescent protein (GFP) and neo (conferring resistance to G418) [19] results in the establishment of latent HHV8 infection, with a very few percentage of cells undergoing lytic replication, a situation thought to mimic in vivo replication. HHV8-infected HMECs exhibited a two to threefold decreased expression of HLA-1 and ICAM-1 compared to uninfected HMECs (Figure 4a left panel), although the remaining expression was still very consistent. We thought that the partial dowmodulation of HLA-1 and ICAM-1 might be explained by the presence of low levels of early lytic immunoregulatory proteins such as K3 or K5 in these latently-infected cells, as previously reported [20], [21]. Indeed, qPCR analysis demonstrated some expression of K3 and K5 mRNAs in infected cells (Figure 4b). In line with the relatively conserved expression of HLA-1 molecules, HHV8-infected HMECs showed a consistent expression of the non-classical HLA class 1b molecule HLA-E, which is dependent on signal sequences from classical HLA-1 molecules for its stabilization [22]. Because the CMV-encoded UL40 protein can also contribute a peptide cargo for HLA-E [23], we searched for potential HLA-E binding peptide motifs in the HHV8 proteins. We did not find any relevant peptide motif, indicating that HHV8 by itself is unlikely to stabilize HLA-E expression (data not shown).

We next analyzed whether HHV8-infected cells expressed ligands for NK cell receptors. We did not observe any expression of NKp30 or NKp46 ligands, or of LLT1, the ligand of CD161, on infected nor uninfected cells. When looking at NKG2D ligands, it appeared that MICA and MICB were up-regulated on HHV8-HMECs compared to uninfected cells, while ULBP-1 was not detected, and ULBP-2 and ULBP-3 were similarly expressed. The ligands of DNAM-1 (CD155/PVR and CD112/Nectin-2) were strongly expressed on both uninfected and infected cells, with a stronger expression of CD155 on HHV8- HMECs.

In attempt to study a model of HHV8-infected cells more clinically relevant to KS than microvascular endothelial cells, we generated an immortalized HIV-negative KS-derived cell line. These SV2G cells exhibited phenotypic characteristics of endothelial cells, as demonstrated by expression of CD146, CD131 and CD141/thrombomodulin (data not shown). However, HHV8 genome was lost early after the first 2 passages, as in most KS-derived cell lines [24]. We thus infected SV2G cells in vitro with rKSHV.152, used above for infecting HMECs. The resulting HHV8-SV2G cell line showed predominantly latent infection, with some expression of K3 and K5 mRNAs, like HHV8-HMECs (Figure 4b). Comparing the phenotype of SV2G and HHV8-SV2G cells, we observed that, for most markers analyzed, modifications paralleled those observed in HHV8-HMEC cells, except for an increased expression of ULBP-2 and ICAM-1 and a weak but significant detection of NKp30 ligand in HHV8-SV2G cells compared to uninfected cells (Figure 4a, right panel).

Collectively, these data show that persistently HHV8-infected cells, which show the same latency program as KS spindle cells, express a variety of ligands that should allow engagement of activating NK cell receptors such as NKG2D, DNAM-1 and NKp30. At the same time however, they show a decreased, but yet strong expression of HLA-1 molecules that likely protects them from NK cell lysis.

To determine whether NK cell receptor/ligand interactions occur at the tumor site, we performed immunohistochemical staining of KS biopsies. In line with the flow cytometric data on infected cells, we readily detected expression of HLA-1 and MICA/B molecules in tumor cells (Figure 4c). We did not observe any staining using the NKp30-Fc or NKp46-Fc reagents. Ligands of DNAM-1 and CD161 could not be analyzed due to the lack of commercially available markers working in paraffin-embedded tissues. To our surprise, although large inflammatory infiltrates were observed in most KS samples, we did not detect any CD56-positive cell, suggesting that NK cells did not reach tumor lesions, or could not survive in the tumor microenvironment.

### NKG2D-mediated NK cell functions are altered in KS patients

**Figure 5:**
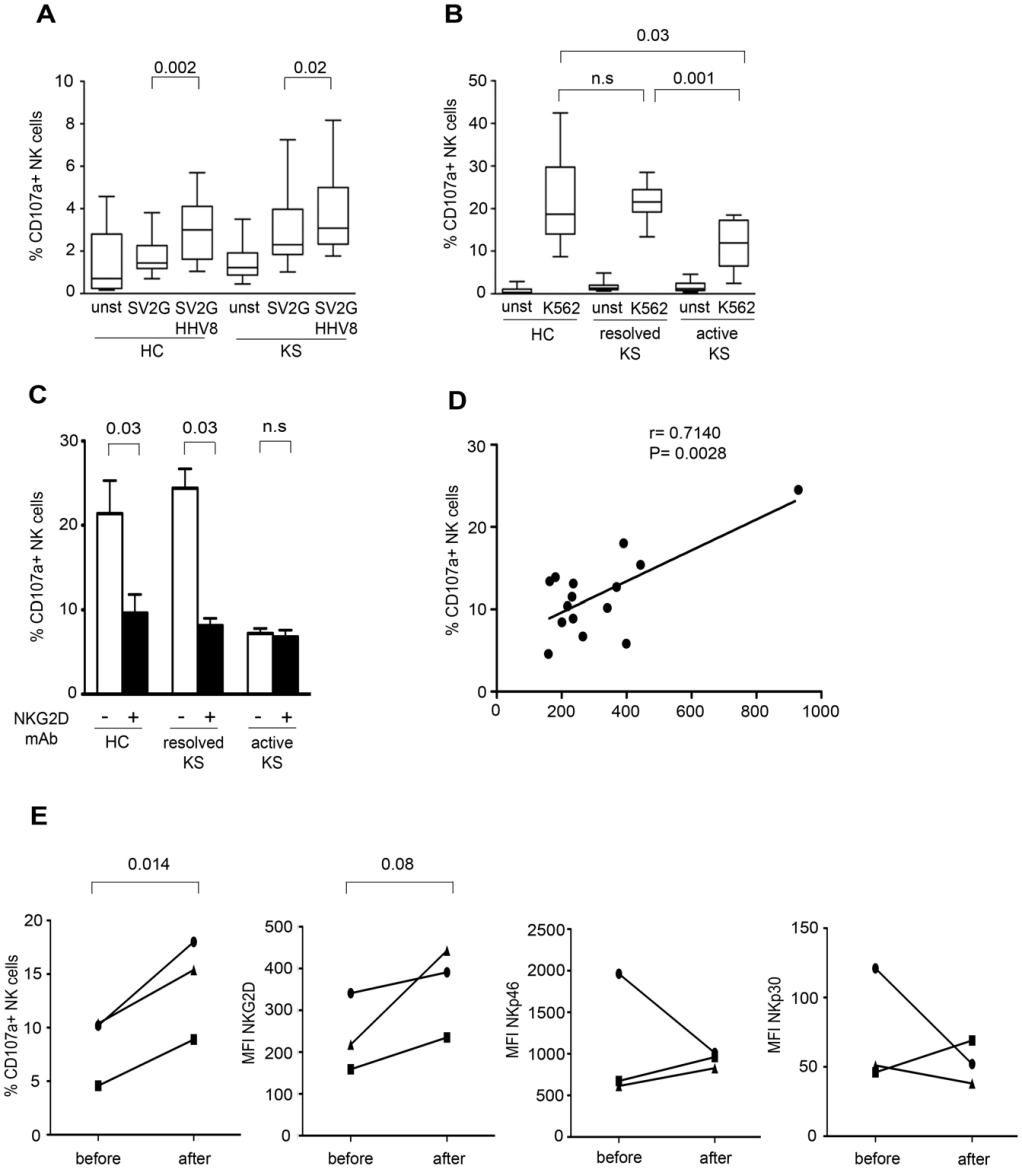
Decreased NK cell lytic capacity in patients with active KS. (A) Comparative analysis of CD107a expression on NK cells from healthy controls (HC, n = 10) and classical KS patients (n = 13, including 3 active and 10 resolved KS). Box and whisker plots show the median and 25–75^th^ percentiles of CD107a expression, in the absence of stimulation (unst) and after stimulation with SV2G or HHV8-SV2G target cells. Horizontal bars indicate minimum and maximum values. (B) The capacity to degranulate in the presence of K562 target cells is significantly decreased in patients with active KS (n = 6) compared to healthy controls (HC, n = 18) and patients with resolved KS (n = 12). (C) K562-induced NK cell degranulation is inhibited in the presence of anti-NKG2D blocking antibody. Data are mean ± SEM of results in 3 healthy controls, 3 patients with resolved classical KS and 2 patients with active classical KS. (D) Correlation between the expression level of NKG2D and the respective K562-induced degranulation of NK cells in 15 KS patients (active/resolved). Cells were analyzed on a LSRFortessa flow cytometer. Correlation coefficient (r) and P values are indicated. (E) Comparative analysis of K562-induced CD107a degranulation and levels of NKG2D, NKp30 and NKp46 in NK cells from 3 patients with active classical KS analyzed before and one year after successful local treatment.

We next evaluated the putative consequences of receptor/ligand modifications on NK cell functions. NK cells from patients with active or resolved classical KS and healthy controls were used as effector cells in CD107a degranulation and intracellular IFNγ production assays in the presence of uninfected or HHV8-infected KS-derived target cells. As anticipated from the relatively strong HLA-1 expression on these target cells, NK cell degranulation was weak and not different between controls and KS patients, whatever the KS activity (Figure 5a). However, degranulation was slightly but significantly higher in the presence of HHV8-infected compared to uninfected targets. Whether this was related to the observed up-regulation of NKG2D, DNAM-1 and NKp30 ligands on infected cells could not be determined. Responses to PMA/ionomycin, used as positive control, were not impaired in patient NK cells (mean CD107a+ cells 40.5% compared to 45.1% in controls), indicating that the granule-exocytosis pathway was intact. Intracellular IFNγ accumulation was almost undetectable in all conditions, both in controls and patients (data not shown).

We then evaluated the ability of NK cells to recognize K562 target cells, which not only lack HLA-1 molecules, but also express ligands for NKG2D, DNAM-1, and NKp30 (personal data and [25]). Compared to healthy controls, patients with resolved classical KS showed intact NK cell degranulation in spite of decreased expression of NKp30, NKp46 and CD161. At contrast, patients with active KS showed significantly impaired NK cell degranulation (Figure 5b), supporting our hypothesis that the NKG2D down-modulation observed in these patients might alter NK cell lytic capacity. Indeed, monoclonal antibody-mediated masking of NKG2D sharply reduced K562-induced degranulation of NK cell from healthy controls and resolved KS patients, but had no effect on NK cells from patients with active KS (Figure 5c). Moreover, we observed a positive correlation between CD107a degranulation and expression of NKG2D (Figure 5d), but not of NKp30 or NKp46. Lastly, comparative analysis of NK cells obtained before and after successful treatment of active classical KS in 3 patients showed that regression of KS was associated with significant restoration of NK cell degranulation, and a parallel increase in expression of NKG2D, but not of NKp30 and NKp46 (Figure 5e).

Taken together, these results suggest that NKG2D may play an important role in the control of KS progression in HHV8-infected individuals.

### PGE2 modulates the NK cell phenotype

Shedding of NKG2D ligands constitutes a major countermechanism of tumors to subvert NKG2D-mediated immunosurveillance. Thus, soluble MICA released from tumor cells by proteolytic cleavage drives down-regulation of NKG2D and is associated with compromised immune response and progression of disease in cancer patients [26]–[28]. We quantified soluble MICA in the serum of KS patients and controls, but found similar low levels in both groups. In addition, we did not detect soluble MICA in culture supernatants of HHV8-infected or uninfected cells (data not shown).

**Figure 6:**
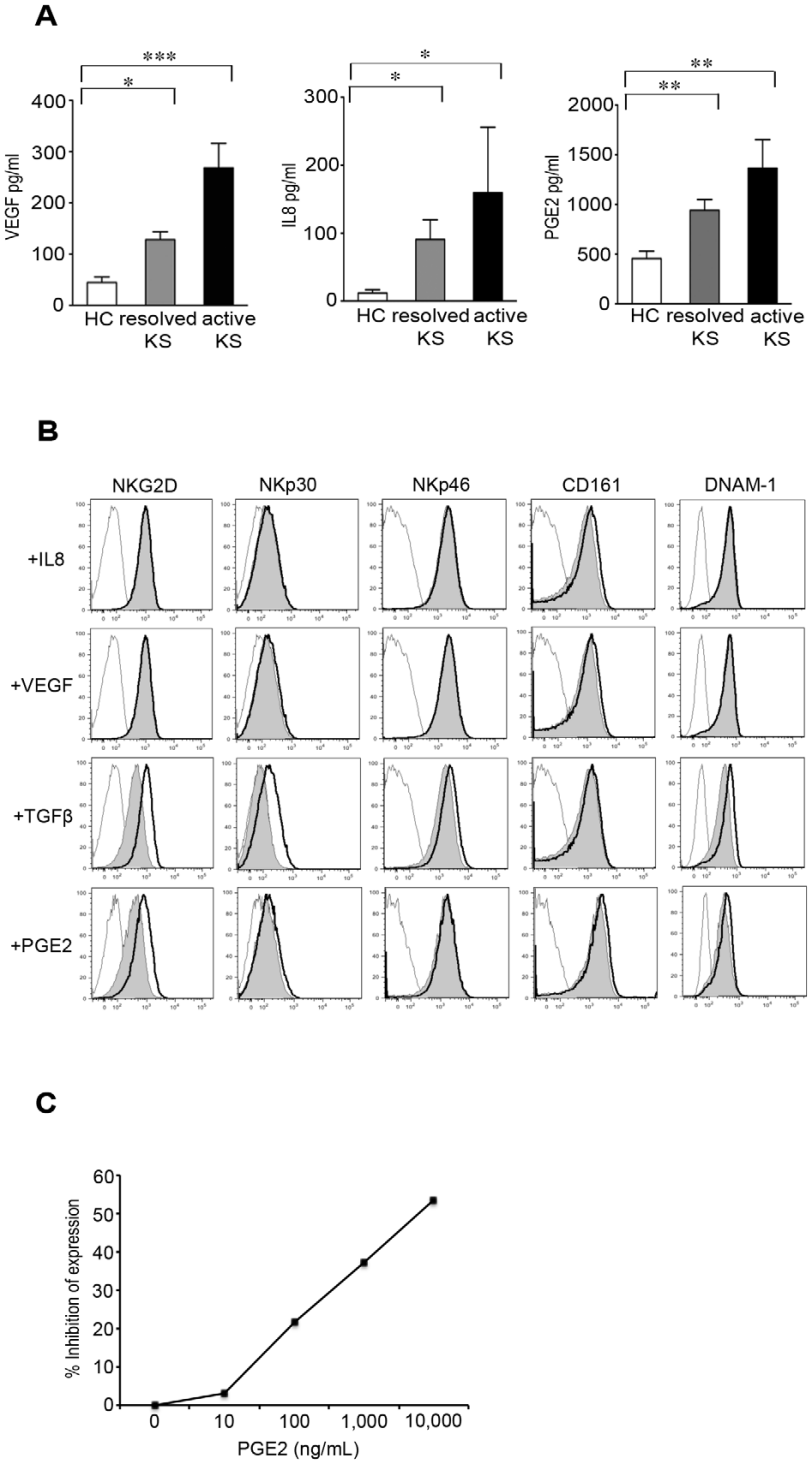
Effect of PGE2 on NK cell phenotype. (A) Levels of VEGF, TGFβ, IL-8 and PGE2 in sera from healthy controls, patients with resolved KS and patients with active KS. P values<0.05 after adjustment for multiple comparisons (Kruskall-Wallis test with Dunn's post test) are indicated. (B) Control PBMCs cells were exposed to IL-8 (100 ng/ml), VEGF (35 ng/ml), TGFβ (10 ng/ml) or PGE2 (100 ng/ml) for 48 h, after which expression of the indicated NK cell receptors was evaluated. Untreated cells are shown as open histograms, and treated cells as gray histograms. Control isotype is shown as thin lines. (C) Dose-dependent PGE2-mediated down-modulation of NKG2D levels. Data are presented as percent of inhibition of NKG2D MFI, and indicate the value means from 3 independent experiments.

HHV8-infected endothelial cells secrete a variety of pro- and anti-inflammatory cytokines, growth factors and angiogenic factors [29]–[31]. In addition, Cyclooxygenase-2 (COX-2) and its metabolite prostaglandin E2 (PGE2), two pivotal proinflammatory molecules, have been shown to play crucial roles in the establishment and maintenance of HHV8 latency, and in inflammatory, angiogenic and invasive events during HHV8 infection [32]–[34]. In attempt to identify the factors responsible for phenotypic changes of NK cells in HHV8-infected patients, we focused on IL-10, TGFβ IL-8, VEGF and PGE2, because they have been involved in the modulation of NK cell functions [35]–[40]. Serum levels of IL-10 and TGFβ were low and not significantly different between patients and controls (data not shown). By contrast, levels of VEGF, IL-8 and PGE2 were significantly increased in KS patients, particularly in those with active classical KS (Figure 6a).

To determine if these factors could modify the NK cell phenotype, control PBMCs were exposed in vitro for 48 h to VEGF, IL-8, or PGE2, after which NK cell receptor expression was evaluated (Figure 6b). TGFβ used as positive control, decreased expression of NKG2D and NKp30 as described [35], but had no significant effect on NKp46, DNAM-1 or CD161. IL-8 and VEGF did not modify the NK cell phenotype. Notably, PGE2 induced a significant decrease of NKG2D expression, but no reproducible modification of the other NK cell receptors. The PGE2-induced down-modulation of NKG2D was dose-dependent (Figure 6c), and was already observed at concentration comparable with those found in the serum of some KS patients. In line with this observation, NKG2D levels on patient NK cells negatively correlated with PGE2 serum levels (r = −0.70, p 0.01).

Taken together, our results suggest that PGE2 may specifically alter NKG2D expression on NK cells, thus preventing NKG2D-mediated elimination of KS cell precursors and favoring the development and/or progression of KS in persistently infected patients.

### PGE2 prevents NK cell responsiveness to IL-15

**Figure 7:**
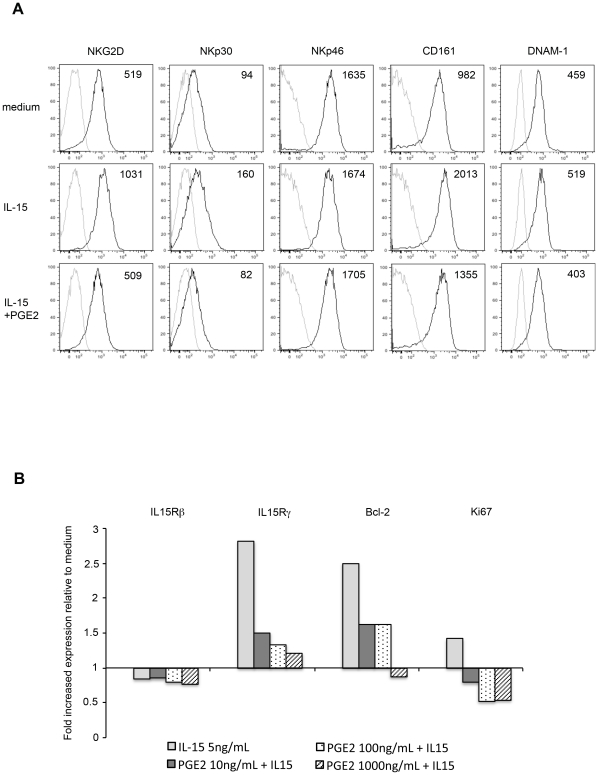
PGE2-mediated suppression of IL-15-induced NK cell. (A) PGE2 inhibits the IL-15-induced up-regulation of NKG2D and NKp30 expression. Freshly isolated PBMCs were treated for 48h in medium alone, or in the presence of IL-15 alone (5 ng/ml), or IL-15 (5 ng/ml) plus PGE2 (100 ng/ml), and analyzed on a LSRFortessa flow cytometer for surface expression of the indicated receptors. Control isotype is shown as thin lines. Values inserted in the histograms indicate MFI and are representative of 3 independent experiments. (B) PGE2 inhibits the responsiveness of NK cells to IL-15. Summarized data show the modification of IL-15Rβ, IL-15Rγ, Ki67 and Bcl-2 expression in the presence of IL-15 alone (light gray) or with increasing concentration of PGE2, relative to medium. Results show the fold increased expression of the indicated marker on NK cells cultured for 48h in the presence of IL-15 and/or PGE2, normalized to the expression on NK cells incubated in medium alone.

IL-15 plays a pivotal role in the activation, function and survival of NK cells. IL-15 is a surface-bound cytokine, presented by dendritic cells via its high-affinity receptor, IL-15Rα to the neighboring NK cells that express IL-2/IL-15Rβ and γ chains. PGE2 has been reported to suppress cytotoxicity of NK cells through down-regulation of IL-15Rγ [39]. Notably, IL-15 is also a potent inducer of NKG2D expression [41], and IL-15 signaling potentiates NKG2D-mediated cytotoxicity of NK cells [42]. To determine if PGE2 could inhibit IL-15-induced up-regulation of NKG2D, control PBMCs exposed for 48 h to 5 ng/ml of IL-15 in the presence or absence of PGE2 (10–1,000 ng/ml), after which NK cell phenotype was analyzed (Figure 7a). IL-15 alone strongly up-regulated expression of NKG2D, NKp30 and CD161, but had no or minor effect on NKp46 and DNAM-1. PGE2, even at low concentration (10 ng/ml), fully abrogated the IL-15-induced up-regulation of NKG2D and NKp30, partially inhibited up-regulation of CD161, and had no effect on expression of NKp46 and DNAM-1.

Because immunochemistry did not detect any CD56-positive cells within KS lesions, we next wondered if PGE2 could mediate a defect in survival of NK cells. We analyzed IL-15-induced NK cell proliferation (expression of Ki67) and survival (expression of the anti-apoptotic protein Bcl-2), together with surface expression of IL-15Rβ and IL-15Rγ (Figure 7b). IL-15Rβ was expressed on almost all NK cells, and was not significantly modified by PGE2. Conversely, PGE2 fully prevented the IL-15-induced up-regulation of IL-15Rγ, as already described. Furthermore, PGE2 was able to abolish the proliferative and pro-survival responsiveness of NK cells to IL-15. Staining for annexin V indicated that PGE2, at the concentrations used (up to 10 µg/ml), did not affect NK cell viability (not shown).

Taken together, our results indicate that PGE2 inhibits IL-15 signaling in NK cells through down-regulation of IL-15Rγ, thus preventing IL-15 from promoting NKG2D signaling.

## Discussion

Infection by the oncogenic virus HHV8 raises issues of control of latent infection and control of tumor growth. HHV8 must overcome host's immune responses not only very early during infection prior to de novo viral gene transcription, but also after latent viral gene expression and later on during tumor transformation. Furthermore, the virus must simultaneously avoid innate and adaptive responses, using strategies that are sometimes mutually exclusive. Multiple evasion mechanisms have been dedicated by herpesviruses to thwart NK cell responses [43]–[45]. They can downmodulate ligands for NK cell activating receptors, provide competitors for their ligands, interfere with their translation, or directly target the activating receptors. It is likely that several viral inhibitor mechanisms play in concert to simultaneously or sequentially prevent NK cell activation. Our study shows for the first time to our knowledge, that distinct NK cell modifications are observed at the different stages of HHV8 infection, suggesting that selective pressure allows the virus to evade the successive waves of host immune responses.

First, asymptomatic HHV8 carriers, as well as KS patients, exhibited significant down-regulation of the NKp30 and NKp46 activating receptors. Whether ligands of these receptors were expressed on HHV8-infected cells could not be ascertained in the absence of specific antibodies, although our data suggest that at least NKp30 ligand was present at the surface of infected cells. Thus, elimination of NKp30 ligand-expressing cells might be compromised in case of NKp30 down-modulation on effector cells. Expression of CD161 was also reduced in asymptomatic HHV8 carriers. This observation was rather surprising, given CD161 is described as an inhibitory receptor in NK cells [46], [47]. We did not evidence expression of its ligand LLT1 on HHV8-infected cells, implying that CD161 may not directly participate in the elimination of these cells. Interestingly, LLT1 is expressed on TLR-activated DCs and B cells [48], suggesting that it may contribute to their resistance to NK cell-mediated lysis. The loss of CD161 might thus result in the accumulation of a population of NK cells with a lower activation threshold that may be more easily triggered, which could lead to the elimination of activated DCs and participate in the defective establishment of antiviral adaptive responses. Because HHV8 infection of monocytes/macrophages and B lymphocytes has been demonstrated in KS patients [49], [50], further studies are required to explore LLT1 expression on these cells, and address whether reduced expression of CD161 modifies NK-DC interactions during HHV8 infection. Notably, expression of DNAM-1, another key receptor in NK-cell mediated recognition of several tumors [51]–[54], was not significantly altered in HHV8-infected individuals. Since DNAM-1 ligands were strongly expressed on HHV8-infected cells, it is possible that at least the DNAM-1 recognition pathway may operate in NK cells during HHV8 infection.

Our observation of a co-modulation of NKp30, NKp46 and CD161 in HHV8 infected subjects suggested us that common microenvironmental factors, acting early during asymptomatic stage of infection, might be involved. Multiple inflammatory and angiogenic factors are produced by HHV8-infected cells. We could not identify a unique factor capable to induce concomitant modifications of these receptors. Although IL-8, VEGF and PGE2 were present in high amounts in patient sera, as reported in AIDS-related KS patients [2], [55], [56], they had no significant effect on expression of these NK cell receptors. TGFβ down-regulated the surface expression of NKp30 as reported [35], [57], but not that of NKp46, or CD161. We cannot exclude that intercellular contact indirectly contribute to alter NK cell phenotype, for instance by inhibiting the functional maturation of DCs, and therefore compromising the DC-NK crosstalk. An effect of the microenvironment on NK cell precursors could also determine their skewed maturation during HHV8 infection, leading to the prevalent expansion of mature NK cells with an altered phenotype. However, we did not observe any skewing in the distribution of CD56bright, CD56dim and CD56-negative NK cell subpopulations, as reported in other chronic infections (HIV, HCV). Finally, the possibility that HHV8 infection of NK cells themselves may modify their phenotype cannot be excluded so far [58]. Various leukocyte subsets support HHV8 latency, including B cells, monocytes, macrophages, DCs and even CD34+ hematopoietic progenitors [49], [50], [59]–[62]. Studies in HHV8-infected NOD/SCID mice demonstrated the presence of LANA+ NK cells in the spleens, suggesting that HHV8 can also target NK cells [63], but this has not been confirmed in the human.

Secondly, in addition to the decreased expression of NKp30, NKp46 and CD161, patients with active classical KS exhibited a specific down-modulation of NKG2D and a parallel defect in NK cell lytic capacity. Resolution of KS after treatment correlated with restoration of NKG2D levels and NK cell activity. A decreased NK cell activity was previously reported in AIDS patients with progressive KS. NK cell function was restored upon ART treatment, but whether this was only related to HIV clearance was not determined [13]. Our results indicate that HHV8 by itself is responsible for the down-modulation of NKG2D in HIV-negative patients with classical KS. Consequently, NKG2D-mediated NK cell cytotoxicity is hampered. A small fraction of KS cells express the cascade of lytic cycle genes, in particular K5, which down-modulates HLA-1, ICAM-1 and certain MICA/B molecules [12]. Loss of surface MICA/B may help HHV8 to evade NK cell surveillance in the early phase of lytic HHV8 infection before establishing latency. This mechanism is clearly not operational in persistently infected KS cells, which express high levels of MICA/B molecules. Instead, the decreased expression of NKG2D appears as an efficient mean for HHV8-infected cells to evade anti-viral immunity and develop their tumoral program. A similar strategy of decreasing NKG2D expression is also adopted by another persistent virus, hepatitis C virus, to evade NK-cell mediated responses in chronically-infected patients [64]. Our results thus reinforce the notion that NKG2D plays an important role not only in control of viral infections, but also in surveillance of tumor development, by protecting the host from tumor initiation and growth [65]–[67].

Soluble NKG2D ligands and TGFβ are known mechanisms for down-regulating NKG2D expression [26], [27] in some cancer patients, but were not involved in KS patients. Chronic expression of NKG2D ligands on tumor tissues also induces the down-regulation of NKG2D [68]. That MICA was strongly expressed in situ within KS lesions may sustain the hypothesis that it is an efficient mechanism to repress antitumor activity by inducing NKG2D down-modulation on intra-tumoral NK cells. However, it does not easily explain why NKG2D was reduced on circulating NK cells. Notably, we observed that PGE2 was able to decrease NKG2D expression on NK cells in vitro, and PGE2 levels in patient sera negatively correlated with NKG2D expression on NK cells.

PGE2 is a major inhibitory factor produced by tumor cells or their surrounding microenvironment [69]. The rate-limiting enzyme in PGE2 synthesis is COX-2, which is over-expressed in many cancers, leading to an over-production of PGE2 often linked to an adverse clinical outcome [70], [71]. COX-2 and PGE2 play crucial roles in the establishment and maintenance of HHV8 latency program [32]. In addition, HHV8-induced PGE2 regulates VEGF, which controls cell growth, adhesion, angiogenesis, proliferation and differentiation. COX-2/PGE2 expression is induced during the early stages of infection of primary HMEC cells [32] and in latently-infected human umbilical vein endothelial cells [34], and abundant COX-2/PGE2 expression is detected in KS tissues [33], [34], [72]. We did not detect the presence of PGE2 in the supernatant of rKSHV.152-infected cells (data not shown), preventing us from reproducing the effect of synthetic PGE2 on NK cells with infected cell supernatants. It must be noted that, although these cells expressed low levels of the early lytic proteins K3 or K5, our attempts to switch latent infection into lytic cycle were always unsuccessful. In KS lesions, KSHV-infected cells show predominantly latent infection, and occasionally undergo lytic reactivation.

Interestingly, PGE2 was shown to downregulate IL-15Rγ chain on NK cells, thus suppressing IL-15-activated NK cell functions [39]. IL-15 is critical for NK cell-dependent clearance of several viral infections, in particular infections by human herpesviruses [73]. Since IL-15 up-regulates expression of NKG2D, and potentiates NKG2D signaling through Jak3-mediated phosphorylation of the NKG2D adaptor DAP10 [41], [42], it is conceivable that PGE2 may profoundly affect NKG2D-dependent NK cell activities. We found that PGE2 not only decreased NKG2D expression on resting NK cells in vitro, but also fully prevented IL-15-induced up-regulation of NKG2D, NKp30 and CD161. Thus, PGE2 overproduction by KS cells may preclude activation of NK cells during HHV8 infection, and promote a progressive drift towards hyporesponsive NK cells. We were surprised by the absence of any CD56+ NK cell within KS lesions, suggesting a defect in homing or survival of NK cells in the vicinity of tumor cells. Indeed, we found that PGE2 inhibited IL-15-induced proliferation and expression of the pro-survival protein Bcl-2 in NK cells, as previously shown in CD4 T cells [74]. Altogether, our results strongly suggest that, by inhibiting IL-15-induced proliferation, activation and NKG2D-mediated function in NK cells, PGE2 appears as a critical factor in preventing immune surveillance of KS development in HHV8-infected individuals. Our results also corroborate recent studies showing the deleterious effect of PGE2 released from mesenchymal stem cells or melanoma-derived fibroblasts on IL-2-induced NK cell activation [37], [75].

In conclusion, our study provides additional clues of the multifactorial complexity of HHV8-host interactions governing KS progression. In addition to previously reported alterations of HHV8-specific CD8 T cell responses in KS patients, we now report how HHV8 stepwise modifies NK cell-mediated activities. These changes may not only affect the early control of HHV8 infection at an asymptomatic stage, but also preclude efficient prevention and immunosurveillance of KS. We also provide new evidence that HHV8 utilizes the inflammatory PGE2 to its advantage in the KS microenvironment, not only for maintaining latency as previously reported, but also for inhibiting NK cell activation, function and survival in response to proinflammatory cytokines.

These results strongly support the potential for COX-2/PGE2 inhibitors in treating KS, as they could simultaneously control latency gene expression and chronic inflammation, reduce angiogenesis and cell adhesion, promote NK cell survival and restore IL-15-induced priming of NKG2D-mediated cytotoxicity.

## Materials and Methods

### Ethics statement

The study was performed in accordance with the Declaration of Helsinki and French legislation, and received approval of the Saint-Louis Hospital Ethical Committee (P040105). All participants provided written informed consent.

### Subjects

The HHV8-infected group consisted of 70 individuals (mean age 56 years), including 25 asymptomatic HHV8 carriers (HHV8+ KS−) and 45 patients with a history of KS (HHV8+ KS+). Because HHV8 infection frequently occurs in the context of HIV infection, patients were sub-classified as follows: HIV− HHV8+ KS− asymptomatic carriers (n = 10, recruited from a cohort of ketosis-prone type 2 diabetes patients [76]); HIV+ HHV8+ KS− asymptomatic carriers (n = 15); HIV− HHV8+ KS+ patients (n = 31 classical KS, including 10 active KS and 21 resolved KS); HIV+ HHV8+ KS+ patients (n = 14 AIDS-related KS, all with resolved KS following antiretroviral therapy). The HHV8-negative control group consisted of 45 age-matched subjects, including 38 healthy blood donor volunteers (HIV− HHV8−), and 7 HIV+ individuals (HIV+ HHV8−). All HIV+ subjects were on stable antiretroviral therapy and had undetectable HIV load for at least 1 year before study. In addition, they were matched for age, CD4 T cell count at time of study, CD4 nadir, duration of disease and duration of ART in the different subgroups. Determination of IgG antibodies against latent and lytic HHV8 antigens was performed by indirect immunofluorescence. Cytomegalovirus (CMV)-specific IgG were detected by ELISA.

### Flow cytometry

Blood samples were processed within 2 h of collection and PBMCs were separated by Lymphoprep gradient centrifugation (Abcys). When indicated, NK cells were freshly purified from PBMCs by negative selection using magnetic microbead separation (StemCell Technologies) with purity higher than 95%. Cells were incubated for 20 min at 4°C with combinations of the following antibodies: FITC-conjugated anti-CD3; PE-conjugated anti-CD56, anti-NKp30, anti-CD158e1e2 (KIR3DL1/S1), anti-CD158i (KIR2DS4); APC-conjugated anti-CD56, anti-CD158ah (KIR2DL1/S1), anti-CD158bbj (KIR2DL2/L3/S2), PE-Cy7-conjugated anti-CD56, Pacific Blue-conjugated anti-CD3 (all from Beckman Coulter); PE-conjugated anti-CD3; PercP-conjugated anti-CD3, APC-conjugated anti-NKp46, anti-CD161; FITC-conjugated anti-CD94, anti-DNAM-1 (BD Pharmingen); PE-conjugated anti-NKG2D (eBioscience); FITC-conjugated anti-CD122 (IL-15Rβ) APC-conjugated anti-NKG2A, anti-NKG2C, anti-CD132 (IL-15RγR&D Systems). For intracellular detection of Ki67 and Bcl-2, cells were fixed in 1% formaldehyde, permeabilized with 0.2% saponin and stained with FITC-conjugated anti-Bcl-2 and PE-conjugated anti-Ki67 (BD Pharmingen). Cells were analyzed on FACSCalibur or LSRFortessa (BD Biosciences), collecting a total of 100,000 events in a live gate. Data were analyzed using FlowJo software.

### HHV8-infected cells

SV40-immortalized human microvascular endothelial cells (HMEC) were infected with rKSHV.152, a recombinant virus expressing the green fluorescent protein (GFP) and neo (conferring resistance to G418), and able to establish cells containing only latent HHV8 [19]. HMEC and HHV8-latently infected HMEC cells (thereafter called HHV8-HMEC) were cultured in MCDB131 medium supplemented with 10 ng/ml epidermal growth factor (EGF) and 1 µg/ml hydrocortisone (Sigma), 10% fetal calf serum (FCS), 2% glutamine, penicillin (100 U/ml) and streptomycin (100 U/ml). In addition, HHV8-HMEC medium contained G418 at 700 µg/ml.

SV2G is an SV40-immortalized HIV-negative KS-derived cell line. Because HHV8 genome was lost early after the first 2 passages, SV2G was also infected in vitro with rKSHV.152. The resulting HHV8-SV2G cells line also showed predominantly latent infection. SV2G cells were cultured in RPMI 1640-10% FCS, while HHV8-SV2G cells were cultured in 50% HMEC medium/50% SV2G medium. Cells were seeded at subconfluent density, and were recovered by trypsine/EDTA treatment. Culture supernatants were collected and kept frozen. Cell viability (ViaProbe, Pharmingen) and phenotype were analyzed by flow cytometry after cell surface staining with antibodies specific for HLA class I (W6/32), MICA and MICB [77]; ULBP-1, ULBP-2, ULBP-3, PVR, LLT1 (all from R&D System), Nectin-2 (BD Pharmingen), HLA-E (clone 3D12, eBioscience) and ICAM-1 (AbD Serotec). Expression of NCR ligands was investigated using NKp30-Fc and NKp46-Fc fusion proteins (R&D) and FITC-anti-human Fc antibody (Jackson Immunoresearch). These reagents were validated for staining NKp30 and NKp46 ligands on K562 and Hela cell lines, previously reported to express these ligands [25], [78], [79].

### NK cell functional assays

Purified NK cells (10^5^ per U-bottom well) were incubated with target cells at 1∶1 effector:target ratio for 6 hr. FITC-conjugated anti-CD107a (20 µg/mL, BD Biosciences) was added directly. After 1 hour at 37°C in 5% CO2, brefeldin A (1 µg/ml) and monensin (6 µg/ml, Sigma) were added for additional 5 hr, and cells were stained with CD56-APC and CD3-PE antibodies, and Viaprobe. Where indicated, NK cells were preincubated with NKG2D blocking antibody (20 µg/ml, Coulter Immunotech) or isotype control. For intracellular IFNγ analysis, cells were fixed following staining with anti-CD3 and anti-CD56, permeabilized with 0.2% saponin and stained with IFNγ FITC antibody (BD) for an additional 30 min.

### Cell cultures and reagents

Recombinant TGFβ, VEGF, IL-8, and IL-15 were purchased from R&D Systems. PGE2 (Cayman Chemicals) was dissolved at 100 mg/ml in 95% ethanol and further diluted with RPMI 1640. The final concentration of ethanol had no effect on NK cell viability and function. ELISA was used to quantify serum IL-8, IL-10, VEGF and TGFβ (R&D Systems), PGE2 (Cayman Chemicals and soluble MICA ([80]).

### Measurement of mRNA levels

Total RNA was extracted from HHV8-infected or uninfected HMEC and KS-derived cells (RNeasy system; Qiagen) and retrotranscribed to cDNA with the use of Superscript III reverse transcriptase and random primers (Invitrogen). For real-time quantitative polymerase chain reaction, Light Cycler 480 SyBR Green I Master and Light Cycler 480 detection system (Roche) were used. The level of K3 and K5 amplified transcripts was determined using a 25-fold dilution of each cDNA, and normalized to glyceraldehyde-3-phosphate dehydrogenase (GAPDH) mRNA levels. Primers used for quantifying the expression of K3 and K5 mRNAs were as follows: 5′-gCAAACCCTgTggAAggATA-3′ (forward) and 5′-AAgCTgCAgggTACAAggAA-3′ (reverse) for K3; 5′-ACCACCACAgACATCAgCAA3′ (forward) and 5′-gTAgggAAgAggTggggAAC-3′ (reverse) for K5.

### Immunohistochemistry

Paraffin-embedded KS biopsy samples from 5 patients were obtained from the Pathology Department. After antigen retrieval in 10 mM citrate buffer pH 6.0 at 100°C, sections were blocked with hydrogen peroxide and PBS containing 10% pooled human AB serum, 1∶30 goat serum and incubated at 20°C for 1 hour with antibodies directed to HLA-1 (W6/32), MICA/B (SR99 [77]) and CD56 (clone 1B6, MBL), or control isotypes, and staining was visualized by using the Envision+ AEC System (Dako). For detection of NCR ligands, sections were incubated with NKp30-Fc or NKp46-Fc fusion proteins (R&D, 8 µg/ml in PBS containing 0.3% normal goat serum), followed by biotin-goat anti-human Fcγ (1∶2,000; Jackson Immunoresearch).

### Statistical analysis

All statistical tests were performed with Instat 3 (GraphPad software). Comparisons between two groups were performed using the Wilcoxon and the Mann-Whitney t tests for paired and unpaired groups, respectively. Multiple comparison analyses were performed using Kruskall-Wallis test (non parametric ANOVA) with Dunn's multiple comparison test. Two-sided p values less than 0.05 were considered significant. Correlation analysis was performed using non parametric Spearman rank correlation.

### Accession numbers

Name: NKG2D, Accession number (Swissprot): P26718, Entry name: NKG2D_HUMAN.

## Supporting Information

Figure S1 FACS gating strategy used to identify CD56-negative NK cells and frequency of this population in the different study groups. Because our initial staining approach did not include anti-CD16 mAb for identification of the CD56-negative CD16+ NK cell populations, we identified NK cells with anti-NKp46 mAb, and characterized the relative frequencies of CD56-negative cells out of total NKp46+ NK cells (A). Representative histograms in a healthy control are shown (B).
(TIF)
