# Supplementary figures and images for: Human Herpesvirus 8 (HHV8) Sequentially Shapes the NK Cell Repertoire during the Course of Asymptomatic Infection and Kaposi Sarcoma
Source: PLoS Pathog. 2012 Jan 12;8(1):e1002486. doi: 10.1371/journal.ppat.1002486 (PMC3257307; doi:10.1371/journal.ppat.1002486)

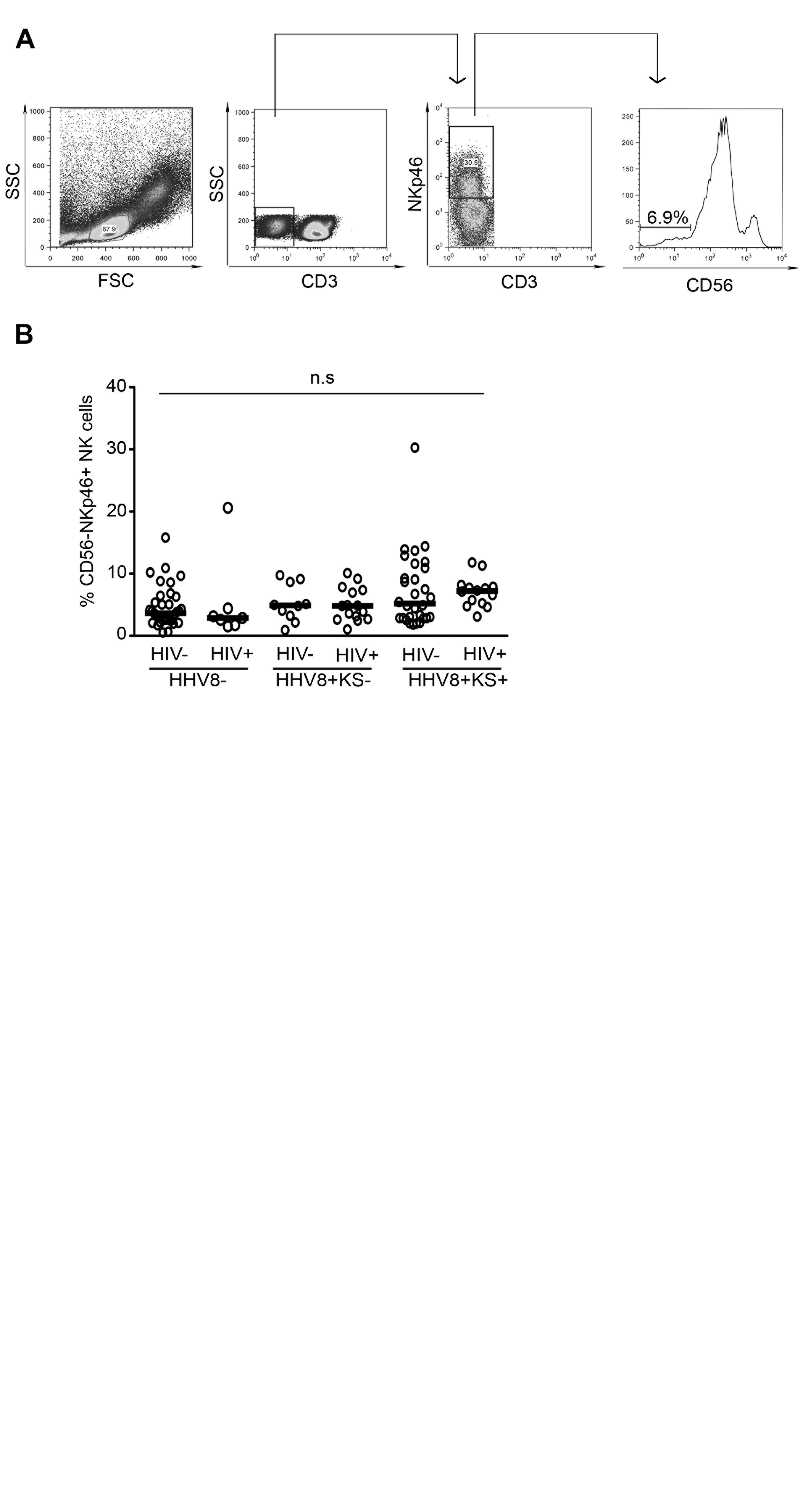

Supplement: Figure S1 — FACS gating strategy used to identify CD56-negative NK cells and frequency of this population in the different study groups. Because our initial staining approach did not include anti-CD16 mAb for identification of the CD56-negative CD16+ NK cell populations, we identified NK cells with anti-NKp46 mAb, and characterized the relative frequencies of CD56-negative cells out of total NKp46+ NK cells (A). Representative histograms in a healthy control are shown (B). (TIF) [file ppat.1002486.s001.tif]
